# 

**Published:** 1891-04

**Authors:** 


### BOOKS AND PAMPLETS RECEIVED.

Sculptured Anthropoid Ape Heads, found near the Valley of the John Day River, Oregon; by *James Terry*. New York, 1891.

Further notes upon the Crania of North American Indians, by *R. A. Shufeldt, M. D., C. M. Z. S.* Reprinted from Journal of Anatomy and Physiology, Vol. xxx.

Memoria de Relaciones exteriores y carteras anexas presentada por el Secretario de Estado Don Ricardo Jimenez. San José de Costa Rica, 1890.

The Journal of the Cincinnati Society of Natural History. Vol. xiii., No. 4.

Bulletin of the American Museum of Natural History. Vol. iii., No. 1. New York.

Annual Report of the Board of Regents of the Smithsonian Institution for the year ending June 30, 1888. Washington 1890.

Transactions of the Canadian Institute. Vol. 1. No. 1. Toronto 1890.

Anales de la Sociedad Cientifica Argentina Tomo xxx. Ent vi. Buenos Aires 1890.

Bulletin de la Societé Zoologique de France Tome xv. No. 10. Paris 1890.

Annalen des K.K. Naturhistorischen Höfmuseums. Band. v. No. 4. Wein 1890.

Bollettino dei Musei di Zoologia ed Anatomia comparata della R. Universita di Torino. Vol. v. No. 74-93.

Bollettino Scientifico Anno xii. No. 4. Pavia 1890.

Der Zoologische Garten. Jahr xxxi. No. 12. Edited by *Dr. F. C. Noll.* Frankfort. AM. 1890.

Mittheilungen des ornithologischen Vereines in Wein. Jahr. xv. No. 2.

Il Naturalista Siciliano. Anno x. No. 2-3. Palermo 1890.

The American Naturalist. Edited by *E. D. Cope* and *J. S. Kingsley.* Published by Ferris Bros., Philadelphia.

Ottawa Naturalist. Vol. iv. No. 11.

Le Naturaliste Canadiene. Cap Rouge, Queebec.

Société Linneennee du Nord de la France. Vol. xix. Nos. 221-2 Amiens.

Special Report on Diseases of the Horse, prepared under the direction of Dr. D. E. Salmon, Chief of the Bureau of Animal Industry. Washington, 1890.

Report of the Commissioners of Fisheries of the State of New York. Albany, 1891.

Archives Neerlandaises des Sciences exactes et naturelles T. xxiv. Liv. Harlem, 1891.

Sitzungs-Berichte der Gesellschaft Naturforschender Freunde zu Berlin 1891.

Journal of the Asiatic Society of Bengal. Vol. lix., pt. 2. Nos. 2, 3, 5. Calcutta, 1890.

Johns Hopkins University Circulars Baltimore, 1891.

Bulletin de L'Academie Royale de Medicine de Belgique, T. v. No. 1. Bruxelles, 1891.

Ornis, edited by Prof. Dr. R. Blasius and Prof. Dr. G. V. Hayke, Jahr vi. Heft 4. Wein 1890.

